# Opposing function of AEBP2 isoforms fine-tune PRC2 catalytic activity

**DOI:** 10.1093/nar/gkag694

**Published:** 2026-07-09

**Authors:** Yingying Li, Cheolan Kwon, Hanbyeol Kim, Chul-Hwan Lee

**Affiliations:** Department of Pharmacology, Seoul National University College of Medicine, Seoul, 03080, Republic of Korea; Department of Biomedical Sciences, Seoul National University College of Medicine, Seoul, 03080, Republic of Korea; Department of Pharmacology, Seoul National University College of Medicine, Seoul, 03080, Republic of Korea; Cancer Research Institute, Ischemic/hypoxic Disease Institute, Neuroscience Research Institute, The Institute of Molecular Biology & Genetics, Seoul National University, Seoul National University, Seoul, 03080, Republic of Korea; Department of Pharmacology, Seoul National University College of Medicine, Seoul, 03080, Republic of Korea; Department of Biomedical Sciences, Seoul National University College of Medicine, Seoul, 03080, Republic of Korea; Department of Pharmacology, Seoul National University College of Medicine, Seoul, 03080, Republic of Korea; Department of Biomedical Sciences, Seoul National University College of Medicine, Seoul, 03080, Republic of Korea; Cancer Research Institute, Ischemic/hypoxic Disease Institute, Neuroscience Research Institute, The Institute of Molecular Biology & Genetics, Seoul National University, Seoul National University, Seoul, 03080, Republic of Korea; Wide River Institute of Immunology, Seoul National University, Hongcheon, 25159, Republic of Korea

## Abstract

Polycomb repressive complex 2 (PRC2) represses genes through catalyzing H3K27me3, a histone modification essential for maintenance of cellular identity. The complex’s catalytic activity, chromatin localization, and propagation along chromatin are modulated by accessory proteins such as AEBP2, MTF2, JARID2, and PALI, which is specifically required for mouse embryogenesis. AEBP2 exists in distinct isoforms: a short isoform that enhances PRC2 catalytic activity and promotes H3K27me3 spreading, facilitating robust gene repression, and a long isoform whose function has remained unclear. Here, we report that the N-terminal region of the long isoform contains conserved DE-motifs unique to this isoform that inhibit PRC2 activity, including both H3K27 methylation and EZH2 automethylation, suggesting that these motifs interfere with the automethylation loop proximal to the SET domain. Notably, re-expression of the long isoform in *Mtf2*/*Jarid2*/*Aebp2* triple-knockout mouse embryonic stem cells failed to restore H3K27me3 and caused defective differentiation. These findings uncover an isoform-specific regulatory mechanism by which AEBP2 controls PRC2 activity and contributes to a broader understanding of the dynamic regulation of PRC2 during development.

## Graphical Abstract

**Graphical Abstract:**
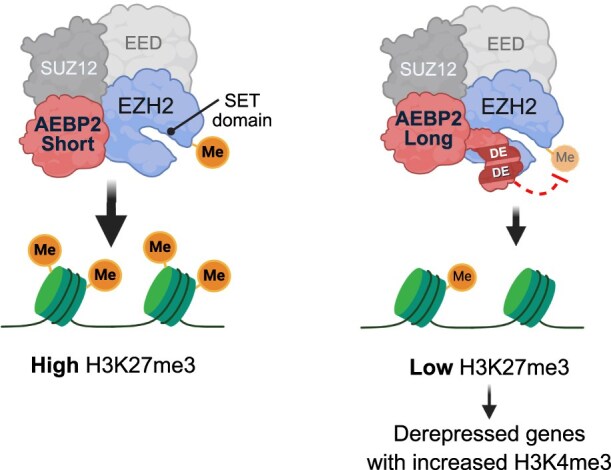

## Introduction

Polycomb repressive complex 2 (PRC2) is a key epigenetic regulator that represses the expression of lineage-specific genes through catalyzing mono-, di-, and tri-methylation on histone H3 lysine 27, H3K27me3 in particular is a hallmark of facultative heterochromatin [1–4]. PRC2 comprises three core subunits: EZH1 or EZH2, alternative catalytic subunits that harbor SET domains; EED, which contains an aromatic cage that can bind to H3K27me3 and allosterically activate PRC2; and SUZ12, which functions as a scaffold protein, interacting with other core subunits and accessory proteins [2, 5–11]. RBAP48 additionally associates with the core subunits to enhance PRC2 functionality, while accessory proteins serve critical functions in recruiting PRC2 to chromatin and enhancing its catalytic activity [7, 12–14]. Based on their accessory components, PRC2 complexes are classified into two major subtypes: PRC2.1, which includes a Polycomb-like protein (either PHF1, MTF2, or PHF19) along with PALI or EPOP; and PRC2.2, which contains JARID2 and AEBP2 [15–18].

The presence and expression of PRC2 accessory proteins is critical for precise control of PRC2 activity in distinct cellular contexts and at specific developmental stages. In mouse embryonic stem cells (mESCs), Mtf2 and Jarid2 are indispensable for the initial recruitment of PRC2 at CpG islands containing GCN- or GA-rich sequences [9]. Previous studies revealed that the JARID2-containing PRC2.2 subcomplex is involved in specific gene regulatory processes during development, contributing to *de novo* formation of H3K27me3 repressive chromatin domains, while the MTF2-containing PRC2.1 subcomplex is responsible for maintaining the pre-existing H3K27me3 repressive domains that are already established in the early embryonic stage [16]. How other PRC2 accessory proteins spatiotemporally regulate PRC2 recruitment and activity remains unclear.

AEBP2 is known to facilitate the interaction between JARID2 and SUZ12, promoting assembly of the PRC2.2 subcomplex [19]. A recent structural investigation has further shown that both AEBP2 and JARID2 interact independently but synergistically with ubiquitinated H2AK119, a critical histone modification that aids in heterochromatin formation [19]. Studies with mice lacking *Aebp2* have demonstrated that its ablation results in later-stage embryonic lethality, while mice having one functional copy (heterozygous deletion) exhibit a variety of abnormalities including enlarged hepatic veins, jugular lymph node sac, enlarged colon, and hypopigmentation. These outcomes highlight the essential role of AEBP2 in organogenesis and tissue specification [17, 18].

AEBP2 is predominantly present in two distinct isoforms, long and short, distinguished by the presence or absence of an acidic-rich N-terminal region [17]. Previous studies have demonstrated that the short isoform enhances the activity of PRC2, specifically its histone methyltransferase (HMT) activity, by promoting nucleosome binding [7]. However, the impact of the long isoform on PRC2 activity remains unclear. Interestingly, the long isoform was identified as the predominant form in adult mouse organs, whereas the short isoform is specifically expressed during the embryonic stage [20]. This indicates that the two isoforms may have distinct functions in different cellular contexts or developmental stages.

In this study, we investigate how the two isoforms of AEBP2, defined by the presence or absence of an N-terminal acidic-rich region, differentially regulate PRC2 catalytic activity, both H3K27 methylation and EZH2 automethylation. We observed that the long isoform, which contains the acidic-rich domain, contributes to inhibition of PRC2 activity, in sharp contrast to the stimulatory effect exerted by the short isoform. By combining *in vitro* reconstitution with cellular assays, we aim to uncover how these opposing influences fine-tune PRC2 function in a context-dependent manner. This work provides a framework to understand how differential expression of AEBP2 isoforms may act as a developmental switch in modulating PRC2-mediated gene repression.

## Materials and methods

### Cell culture

mESCs (E14) and derivates were grown on 0.5% gelatin-coated dishes in Dulbecco’s modified Eagle’s medium containing 15% (v/v) fetal bovine serum (Biowest, 51480-500), penicillin streptomycin (10,000 U/ml), 2 mM L-Glutamine, 1× MEM non-essential amino acids, 10 mM 2-Mercaptoethanol and mouse Leukemia Inhibitory Factor (LIF, ESGRO^®^, ESG1107), 1 mM MEK1/2 inhibitor (Tocris, 4192), and 3 mM GSK3 inhibitor (Tocris, 4423) at 37°C with 5% CO_2_.

### Recombinant bacterial protein

Human AEBP2 long (isoform 1 in human, a.a. 1~503), short (a.a. 219~503), and truncated forms (T1, a.a 75~503, and T2, a.a 145~503) were cloned into pET21a. Strep-tagged AEBP2 long, short, and truncated forms were expressed in *Escherichia coli* strain Rosetta with LB media for 16 h at 18°C by induction with 0.5 mM isopropyl β-d-1-thiogalactopyranoside. *Escherichia coli* cells were resuspended in 20 mM Tris–HCl (pH 7.5), 350 mM NaCl, 10% glycerol, and protease inhibitor [1 mM phenylmethylsulfonyl fluoride (PMSF), 1 mM benzamidine, 1 μg/ml leupeptin, and 1 μg/ml pepstatin]. Cell lysate was purified using Streptactin Sepharose Beads (Cytiva) for 3 h at 4°C and eluted by desbiotin, and the proteins were again purified with Q Sepharose Beads (Cytiva). Purified proteins were dialyzed with 20 mM Tris–HCl (pH 7.5), 350 mM NaCl, and 10% glycerol.

### Protein purification using baculovirus expression system

Baculovirus expression plasmids for human EZH2, SUZ12, Flag-tagged EED, and RBAP48 were cloned into pFastBac1. The recombinant pFastBac1 vectors were transformed to *E. coli* DH10Bac cells to create recombinant bacmid. Recombinant human PRC2 core subunits (EZH2, EED, SUZ12, and RBAP48) were co-expressed in Sf9 insect cells grown in SFM Media (Expression System). After 64 h of infection, cells were resuspended in 20 mM Hepes–HCl (pH 7.5), 350 mM NaCl, 10% glycerol, and protease inhibitor (see above) and lysed with a cell disruptor (machine name). Then, PRC2 complex was purified using FLAG-M2 Agarose Beads (Sigma) and Q Sepharose (Cytiva). Purified proteins were dialyzed with 20 mM Tris–HCl (pH 7.5), 180 mM NaCl, and 10% glycerol. For generating PRC2–AEBP2 complexes, AEBP2 long, short, and truncated forms purified from the *E. coli* system were incubated with PRC2 Sf9 cell lysate before purification. The purification step was the same as the PRC2 core complex purification mentioned above.

### Nucleosome reconstitution

Recombinant histones were generated as previously described [21]. Briefly, each histone was expressed in Rosetta (DE3) cells (Novagen), extracted from inclusion bodies, and purified by sequential anion chromatography and size exclusion chromatography. For refolding recombinant octamers, equimolar amounts of histones were mixed and dialyzed into refolding buffer [10 mM Tris–HCl, pH 7.5, 2 M NaCl, 1 mM ethylenediaminetetraacetic acid (EDTA), and 5 mM β-mercaptoethanol]. Octamers were further purified by size exclusion chromatography on a Superdex 200 Column (GE Healthcare) in refolding buffer. Recombinant oligonucleosomes were reconstituted by gradient salt dialysis of octamers and indicated DNA fragments or Cy5-labeled 601-nucleosome positioning sequences.

### Methyltransferase assay

Standard methyltransferase assays were performed in a total volume of 15 ml containing HMT buffer (50 mM Tris–HCl, pH 8.5, 5 mM MgCl_2_, and 4 mM 1,4-Dithiothreitol) with either 50 mM of cold S-Adenosylmethionine (SAM, Merck) or 1 μM Adenosyl-L-Methionine, S-[methyl-3H]- (Revvity), 100 nM of nucleosomes, and recombinant human PRC2 complexes under the following conditions. The reaction mixture was incubated for 60 min at 30°C and stopped by adding 4 μl of STOP buffer (0.2 M Tris–HCl, pH 6.8, 20% glycerol, 10% sodium dodecyl sulfate (SDS), 10 mM β-mercaptoethanol, and 0.05% bromophenol blue). The yield of each methyltransferase reaction was measured as follows: after the reactions, samples were incubated at 95°C for 5 min and then separated using sodium dodecyl sulfate–polyacrylamide gel electrophoresis (SDS–PAGE). Proteins were transferred to 0.45 µm PVDF membranes (Millipore) via wet transfer. For reactions using cold SAM, the H3K27me3 levels were detected by western blot using an H3K27me3-specific antibody. For reactions using hot SAM, the total levels of H3K27 methylation and EZH2 methylation were assessed by autoradiography [22].

### Gel mobility shift assay

Cy5-labeled core nucleosomes were incubated with PRC2 or PRC2–AEBP2 complexes in 10 mM HEPES, pH 7.9, 50 mM KCl, 1 mM glycerol in a total volume of 15 μl for 30 min at room temperature (RT). Complex formation was analyzed by 4% native polyacrylamide gel electrophoresis (0.3 × Tris–borate–EDTA, 5% glycerol). Cy5 signal was detected using the ChemiDoc™ MP Imaging System (Bio-Rad Laboratories, USA).

### Preparation of whole cell extracts and western blotting

Cells were harvested and lysed with RIPA buffer (10 mM Tris–HCl, pH 7.5, 400 mM NaCl, 1 mM EDTA, 1% Trition X-100, 0.1% sodium deoxycholate, and 0.1% SDS) containing protease inhibitors (0.5 mM PMSF, 1 mM benzamidine–HCl, 1 μg/ml pepstatin, 1 μg/ml leupeptin) and phosphatase inhibitors (10 mM NaF and 1 mM Na_3_VO_4_). The cell suspension was sonicated (40% amplitude, 12 strokes) and centrifuged at 20,000 × *g* at 4°C for 10 min. The supernatant (whole cell extract) was mixed with denaturing sample buffer, boiled for 5 min and run onto 4%–16% SDS-PAGE gels. The gels were transferred onto a PVDF membrane at 100 V for 90 min. PDVF is blocked in TBS-T-milk for 1 h at RT, then O/N at 4°C with primary antibody and 1 h at RT with secondary antibody.

The following antibodies were used: H3 (1:1000, #A01100, #ATSJL3001, Abbkine), H3K27me1 (1:1000, #84932S, #1, Cell Signaling Technology), H3K27me2 (1:1000, #9728S, #16, Cell Signaling Technology), H3K27me3 (1:1000, #9733S, #27, Cell Signaling Technology), H3K4me3 (1:1000, #9751S, #16, Cell Signaling Technology), β-tubulin (1:10 000, #ABL1030, #ATXI24021, Abbkine), β-actin (1:10 000, #A01010, #ATUJA1301, Abbkine), AEBP2 (1:1000, #14129S, #1, Cell Signaling Technology), JARID2 (1:1000, #13594S, #3, Cell Signaling Technology), MTF2 (1:1000, #16208-1-AP, #N/A, Proteintech), EZH2 (1:1000, #5246S, #12, Cell Signaling Technology), SUZ12 (1:1000, #3737S, #10, Cell Signaling Technology), SOX2 (1:1000, #2748S, #4, Cell Signaling Technology), NANOG (1:2000, #4903S, #8, Cell Signaling Technology), and OCT4 (1:1000, #sc-5279, #G0121, Santa Cruz Biotechnology), EZH2-K514me3 [23, 24].

### Lentiviral production and delivery

AEBP2 short isoform, long isoform, and truncated fragments of the long isoform (T1 and T2, respectively) were subcloned into the pLV-EF1-alpha-IRES-Puro vector (Clontech). Subcloned lentiviral vectors were co-transfected with pcREV, BH-10, and pVSV-G packaging vectors (Viral vector 10 mg, VSVG 3 mg, BH10 5 mg, and pcREV 2.5 mg) into Lenti-X 293T cells to produce viral particles. Virus-containing medium was collected 48 h after transfection and polybrene was added to the viral medium at a concentration of 8 μg/ml. The target cells were infected and sorted for mCherry positive by FACS cytometry (BD FACSAria™ III).

### Generation of CRISPR/Cas9-mediated genome editing cell lines

*Aebp2* KO (AKO) cell line was obtained from a previous study [7]. The *Aebp2, Mtf2*, and *Jarid2* triple-knockout (TKO) mESC line was generated by sequentially introducing *Mtf2* and *Jarid2* deletions into the AKO background, following previously published methods [8, 9]. Oligonucleotides encoding each sgRNA were cloned into the SpCas9-2A Puro (Addgene: PX459) via BbsI digestion and insertion. mESC cells were seeded into 6-well plates at 80,000 cells per well and transfected with 1.5 μg of the appropriate guide RNAs, template DNA for guide RNAs and Cas9 endonuclease using Lipofectamine 2000 (Thermofisher). The transfection was performed using a 2:1 ratio of Lipofectamine to DNA. After transfection, cells were selected with 2 μg/ml puromycin for 48 h and single ESC clones were picked after 7–10 days, trypsinized in 0.25% Trypsin-EDTA for 5 min, and plated into individual wells of a 96-well plate for genotyping. Each clone was characterized to confirm gene knockout by Western-Blot and gDNA sequencing.

### CUT&Tag

Cells were collected and washed twice with PBS, then fixed with 0.1% formaldehyde for 1 min. The fixation was quenched by adding 0.125 M glycine, followed by another PBS wash. The cells were resuspended in Nuclear Extraction (NE) buffer (20 mM HEPES, pH 7.9, 10 mM KCl, 0.1% Triton X-100, and 20% glycerol) and incubated on ice for 10 min. Following nuclear extraction, the nuclei were washed with cold NE buffer and resuspended in cold NE buffer at a concentration of 0.05 million nuclei/100 μl. The nuclei were then immobilized on Concanavalin A Conjugated Paramagnetic Beads (Epicypher, #21-1401). After immobilization, the nuclei were permeabilized with 0.01% digitonin and incubated overnight at 4°C with the primary antibodies (1 μl/reaction): H3K27me3 (Cell Signaling Technology, #9733) or H3K4me3 (Cell Signaling Technology, #9751). The CUT&Tag-IT Spike-In Control (Active Motif, #53168) was included in parallel following the manufacturer’s protocol to allow normalization of signal intensity across samples. Afterward, samples were incubated with 0.5 µg of anti-rabbit secondary antibody (Epicypher, #13-1047) for 1 h. Following the secondary antibody incubation, pA-Tn5 (Epicypher, #15-1017) was added, and the samples were incubated for 1 h at RT. Targeted tagmentation was mediated by adding magnesium chloride (10mM MgCl_2_) and the reactions were incubated at 37°C for 1 h. After tagmentation, the reaction was stopped, and the DNA fragments were amplified using PCR. The libraries were quantified, quality-checked, and sequenced on an Illumina NovaSeq X Plus platform using 150 bp paired-end reads.

### ChIP-seq

For double crosslinking, 3 × 10^6^ cells were harvested and fixed sequentially with 2 mM disuccinimidyl glutarate (Thermo Fisher, #20593) for 35 min, followed by 1% formaldehyde for 10 min at RT. Crosslinking was quenched with 0.125 M glycine, and nuclei were isolated in NE buffer and extracted nuclei were then resuspended 1% SDS lysis buffer (50 mM Tris-HCl pH 8.0, 10 mM EDTA pH 8.0, 1% SDS) and sonicated by Covaris S220 sonicator (Power 175 W, Duty 10%, 200 cycles per burst) for 430 sec to generate DNA fragments of ~200 bp. Sheared chromatin was diluted in ChIP dilution buffer (16.7 mM Tris-HCl, pH 8.0, 0.01% SDS, 1.1% Triton X-100, 16.7 mM NaCl, 1.2 mM EDTA) and clarified by centrifugation at 13,000 rpm for 15 min at 4°C.

Chromatin was incubated overnight at 4°C with Dynabeads protein A pre-bound to 2.4 μg of target antibodies, including AEBP2 (Cell Signaling Technology, #14129S) and SUZ12 (Cell Signaling Technology, #3737S). 1 μg of Drosophila chromatin (Active Motif, #53083) and 0.1 μg of anti-Drosophila H2A.X antibody (Active Motif, #61686) were added per reaction to perform spike-in normalization. The next day, Dynabeads were washed three times with buffer A (140 mM NaCl, 1 mM EDTA pH 8.0, 0.5 mM EGTA pH 8.0, 1% Triton X-100, 0.1% SDS, 0.1% sodium deoxycholate, 10 mM Tris-HCl pH 8.0), twice with buffer B (300 mM NaCl, 1 mM EDTA pH 8.0, 0.5 mM EGTA pH 8.0, 1% Triton X-100, 0.2% SDS, 0.1% sodium deoxycholate, 10 mM Tris-HCl pH 8.0) and once with buffer C (250 mM LiCl, 10 mM Tris-HCl pH 8.0, 1 mM EDTA, 0.5% NP-40, 0.5% sodium deoxycholate). RNA was removed by treatment with RNase A at 37°C for 30 min and crosslinks were reversed overnight at 65°C in the presence of Proteinase K and SDS treatment.

Eluted DNAs were size-selected using SPRIselect beads (Beckman, #B23319) and libraries were prepared with NEB DNA library prep kit (NEB, #E7645L) following the manufacturer’s protocol. The libraries were quantified, quality-checked, and sequenced on an Illumina NovaSeq X Plus platform using 150 bp paired-end reads.

### ChIP-seq and CUT&Tag analysis

Adaptor sequences were trimmed using Cutadapt, and the filtered reads were aligned to the mouse reference genome (mm10) with Bowtie2 using default parameters. PCR duplicates were identified and removed with SAMtools markdup. Unaligned reads were subsequently mapped to the *Drosophila melanogaster* reference genome (dm6), and the number of Drosophila-mapped reads was used to calculate the spike-in normalization factor. Normalized bigWig files, heatmaps, and average profiles were generated using deepTools. For peak calling, ChIP-seq data were processed using MACS2 with IgG controls and broad peak calling parameters. CUT&Tag data were also subjected to broad peak calling using MACS2.

To evaluate the effects of AEBP2 isoforms on H3K27me3 deposition, we performed a genome-wide bin-based analysis. Briefly, normalized CUT&Tag signals were summarized into non-overlapping 10-kb bins across the mouse genome using deepTools. To focus on regions with robust PRC2 activity, bins corresponding to the upper quantile (≥0.9 quantile) of H3K27me3 signal in wild-type (WT) samples were selected. These bins were then used to assess changes in H3K27me3 levels as well as AEBP2 and Suz12 occupancy across different conditions. Pairwise relationships between signals were visualized using density scatter plots, and distributions of signal intensities were compared using box plots.

### RNA-seq

A total of 5 × 10⁶ cells were harvested and resuspended in 1 ml of TRIzol reagent, followed by incubation for 5 min at RT. Chloroform (0.2 ml) was added, and the samples were vortexed vigorously and centrifuged at 13,000 rpm for 15 min at 4°C. The upper aqueous phase containing RNA was transferred to a new tube, and RNA was precipitated with isopropanol and washed with ice-cold 70% ethanol. The RNA pellet was air-dried, dissolved in DEPC-treated water (Invitrogen, #AM9915G), and its concentration and integrity were assessed. Sequencing libraries were prepared using the TruSeq Stranded Total RNA Ribo-Zero Gold Kit (Human/Mouse/Rat) (Illumina, #RS-122-2301). The libraries were then sequenced on an Illumina NovaSeq X Plus platform to generate 150 bp paired-end reads.

### RNA-seq analysis

Adaptor sequences were trimmed using trimmomatic, and the filtered reads were aligned to the mouse reference genome (mm10) using HISAT2. Transcript assembly and quantification were performed using StringTie, and gene-level read counts were obtained with featureCounts. Genes with zero total counts across all samples were removed prior to downstream analyses. Normalization factors were estimated using the median-of-ratios method implemented in DESeq2. Differential expression analysis was carried out using DESeq2 (Wald test), and genes with an adjusted *P*-value <.05 and |log_2_ fold change| > 2 (or 1, where indicated) were considered significantly differentially expressed. Gene Ontology (GO) and pathway enrichment analyses were performed using the DAVID. Visualization of normalized expression levels and heatmaps was conducted in R (v4.2.3) using ggplot2 and pheatmap. For visualization, normalized bigWig files were generated from BAM files using deepTools with counts per million normalization and a bin size of 10 bp. Genome browser tracks were visualized using IGV. GO enrichment analysis (GOTERM_BP_FAT) was performed using the Database for Annotation, Visualization, and Integrated Discovery (DAVID). Significantly enriched GO terms were visualized as bar plots in R (v4.2.3) using ggplot2. Visualization of normalized expression levels and Venn diagrams was conducted in R (v4.2.3) using ggplot2.

### mESC differentiation

Approximately 1 × 10⁵ mESCs were plated on 60-mm dishes, and differentiation was induced by withdrawal of LIF, PD0325901, and CHIR99021 from complete medium. Medium was replaced daily, and cells were harvested after 4 days of LIF/2i removal.

### Subcellular protein fractionation

Subcellular protein fractionation was performed using the Subcellular Protein Fractionation Kit for Cultured Cells (Thermo Fisher, #78840) according to the manufacturer’s protocol. The resulting cytoplasmic, nuclear, and chromatin fractions were analyzed by western blotting using standard procedures.

## Results

### The AEBP2 long isoform inhibits PRC2 activity while the short isoform stimulates it

**Figure 1.**
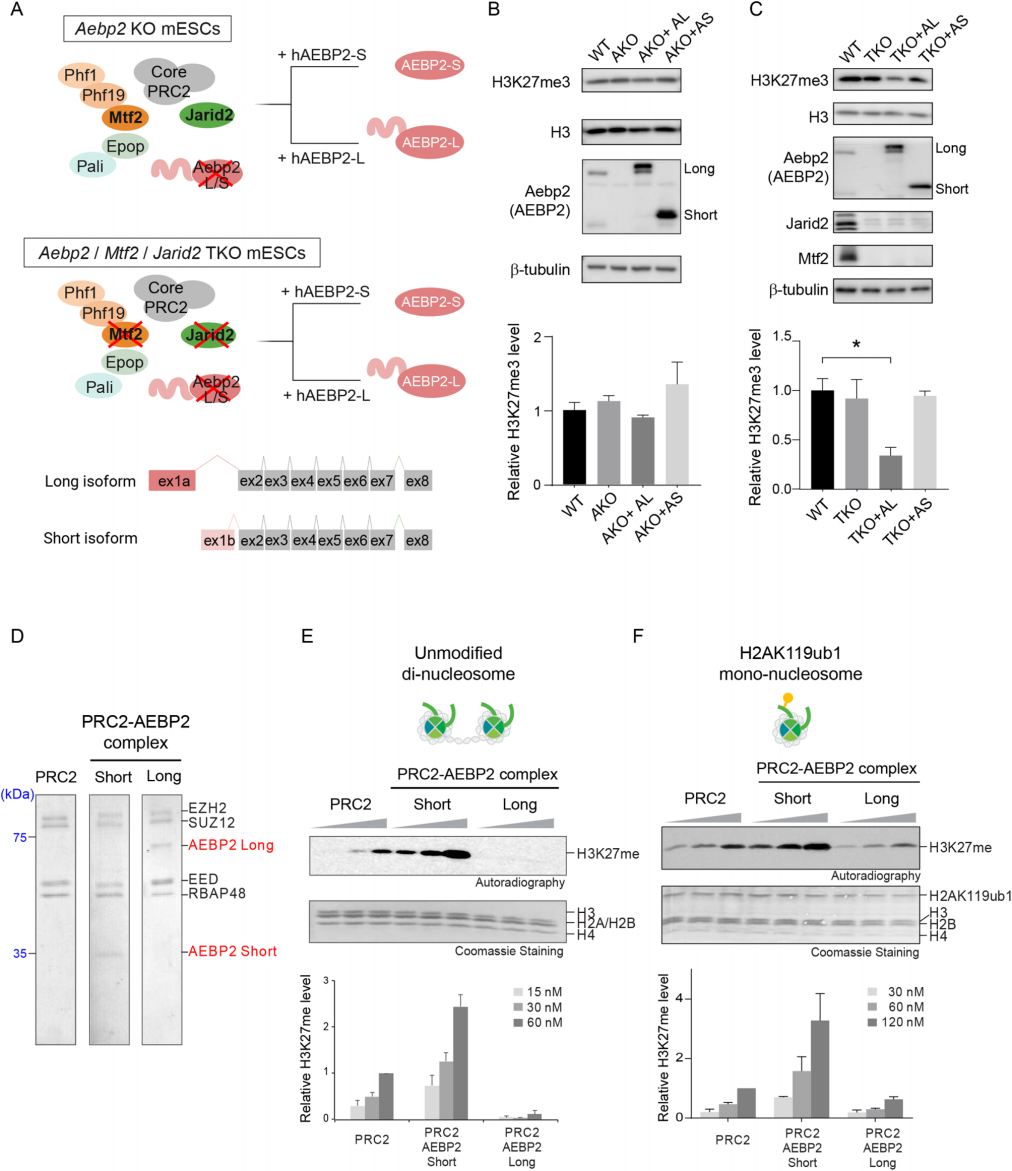
The AEBP2 short isoform stimulates PRC2 activity, whereas the long isoform inhibits it. (**A**) Schematic of AKO or *Aebp2/Mtf2*/*Jarid2* TKO mESCs, followed by rescue with human AEBP2, either short or long isoform. The two key accessory proteins responsible for PRC2 recruitment in mESCs, Mtf2 and Jarid2, were emphasized with bold outlining (top). Schematic representation of *AEBP2* gene structure showing alternative transcription start sites (TSS). The long isoform initiates from exon 1a, while the short isoform originates from exon 1b, respectively, resulting in proteins of ~53 kDa (AEBP2 long isoform) and 31 kDa (AEBP2 short isoform) (bottom). Created in BioRender, Lee, C. (2026) https://BioRender.com/mpilqw5. (**B**) Western blot analysis of H3K27me3, H3, Aebp2 (AEBP2), and β-tubulin in WT, AKO, or AKO mESCs rescued with either AEBP2 long isoform (AL) or AEBP2 short isoform (AS) (top). Quantification of H3K27me3 is shown (*n* = 3 per data point; mean ± SD) (bottom). (**C**) Western blot analysis of H3K27me3, H3, Aebp2 (AEBP2), Jarid2, Mtf2, and β-tubulin in WT, TKO, and TKO mESCs rescued with specific AEBP2 isoforms (top). Quantification of H3K27me3 is shown (*n* = 3 per data point; mean ± SD) (bottom). (**D**) Coomassie staining of purified PRC2 or PRC2 in complex with long or short isoform of AEBP2. (**E**) HMT assay containing PRC2 or PRC2 in complex with short or long isoform (15, 30, 60 nM) using unmodified di-nucleosomes (200 nM) as substrate. H3K27 methylation level was determined by autoradiography (top), and relative quantification compared after 60 min of incubation is shown (*n* = 3/data point) (bottom). (**F**) HMT assay of PRC2 or PRC2 in complex with AEBP2 short or long isoform (30, 60, 120 nM) using H2AK119ub1 mono-nucleosomes (200 nM) as substrate. H3K27 methylation level was determined by autoradiography (top), and relative quantification compared after 60 min of incubation is shown (*n* = 3/data point) (bottom).

The long and short isoforms of AEBP2 originate from the use of alternative start exons (Supplementary Fig. S1A–C). To investigate how each isoform differentially modulates PRC2 activity, either the short isoform or the long isoform was ectopically expressed in AKO mESCs (Fig. 1A). Expression of the long isoform resulted in a modest reduction in global H3K27me3 levels (Fig. 1B), consistent with recent reports suggesting an inhibitory role of the long isoform in PRC2 regulation [25].

Given that PRC2 recruitment in mESCs is primarily mediated by Mtf2 and Jarid2 [9], and that both factors are highly expressed at the mESC stage [26] (Supplementary Fig. S1D and E), their presence may mask the functional consequences of AEBP2 loss. To more directly assess the intrinsic effects of AEBP2 isoforms on PRC2 activity, we therefore extended our analysis using TKO mESCs lacking *Aebp2, Jarid2*, and *Mtf2* (Fig. 1A). In this context, reintroduction of the AEBP2 long isoform led to a pronounced decrease in H3K27me3 (Fig. 1C), revealing a stronger inhibitory effect in the absence of canonical recruitment pathways. These findings suggest that, unlike the previously characterized short isoform [7], the long isoform of AEBP2 can act as a negative regulator of PRC2 activity. In addition, the TKO cells did not exhibit a substantial reduction in H3K27me3, likely because other PRC2 accessory proteins, such as Epop, Phf19, and Pali, can compensate for the loss of Aebp2, Jarid2, and Mtf2. However, this compensation was not observed when the TKO cells were rescued with the AEBP2 long isoform, suggesting that the long isoform exerts a dominant inhibitory effect over the remaining accessory proteins.

To directly compare PRC2 activity, we purified human PRC2 reconstituted with either the long or short isoform together with the core subunits (EED, SUZ12, and EZH2) and RBAP48 (Fig. 1D). A subsequent HMT assay using unmodified di-nucleosome showed the short isoform to stimulate PRC2 activity while the long isoform inhibited it (Fig. 1E). The latter inhibitory effect was also observed when using H2AK119ub1-modified mono-nucleosome (Fig. 1F), which is known to interact with AEBP2 through its Zn-finger domain [19].

### The acidic DE-rich motifs exclusively on the N-terminal region of the AEBP2 long isoform are essential for its inhibitory effect on PRC2 activity

**Figure 2.**
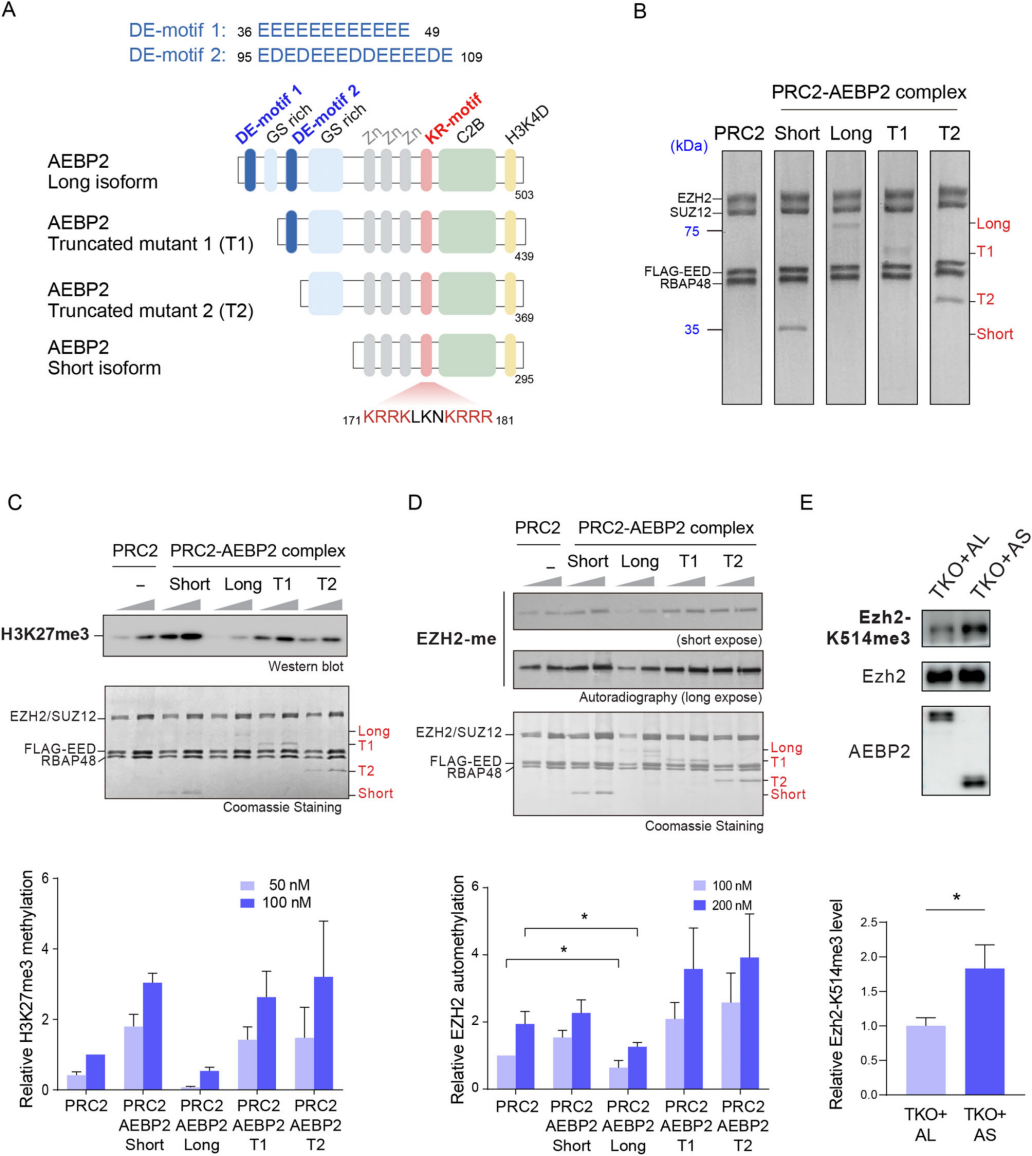
The AEBP2 long isoform inhibits both EZH2 auto-methylation and H3K27 methylation through DE-motifs in its N-terminal. (**A**) Schematic representation of AEBP2 full-length short isoform, long isoform, and truncated fragments (T1 and T2). The KR motif, required for the short isoform to enhance nucleosome binding and stimulate PRC2, is detailed below; sequences of DE-rich motifs are shown above. Created in BioRender, Lee, C. (2026) https://BioRender.com/a52mlnv. (**B**) Coomassie staining of purified PRC2 and respective PRC2–AEBP2 complexes incorporating. (**C**) HMT assay of PRC2 or PRC2–AEBP2 complexes (50, 100 nM) using di-nucleosomes (150 nM) as substrate. H3K27 methylation was determined by western blot with H3K27me3 antibody (top), and relative quantification compared after 60 min of incubation is shown (*n* = 3/data point) (bottom). (**D**) Methyltransferase assay containing PRC2 or PRC2–AEBP2 complexes (50, 100 nM) with ^3^H-labeled SAM. Autoradiography shows that the long isoform and its DE-rich fragments inhibit EZH2 automethylation (top), and the relative amount of EZH2 automethylation was quantified after 60 min of incubation (*n* = 3/data point) (bottom). Statistical significance was determined using an unpaired two-tailed Student’s *t*-test; *P < .05. (**E**) Western blot analysis of Ezh2-K514me3 in nuclear extracts of TKO + AL and TKO + AS cells. Relative Ezh2-K514me3 levels normalized to Ezh2 are shown (bottom). Data are presented as mean ± SD (*n* = 3). Statistical significance was determined using an unpaired two-tailed Student’s *t*-test; *P < .05.

To gain mechanistic insight into how AEBP2 isoforms differentially regulate PRC2 activity, we next examined their amino acid sequences, focusing on the N-terminal region unique to the long isoform. The long isoform possesses an additional N-terminal region with 208 amino acids, including two negatively charged DE-motifs (Fig. 2A and Supplementary Fig. S2A). These DE motifs are predicted to interact with positively charged regions of PRC2 and influence complex activity. Our previous studies have also shown the KR-motif in AEBP2 to be crucial for enhancing nucleosome binding and stimulating PRC2 activity [7]. We therefore hypothesized that the DE-rich motifs may prevent interaction of the KR-motif with nucleosomal DNA or with other positively charged regions within PRC2, ultimately attenuating catalytic activity.

To test this hypothesis, we generated truncation mutants of AEBP2 that remove individual DE-rich regions (Fig. 2A). The T1 mutant removes the N-terminal portion containing DE-motif 1 but retains DE-motif 2, whereas the T2 mutant truncates further downstream and removes both DE-rich motifs; in both cases, the truncation points are positioned within GS-rich regions (Fig. 2A and Supplementary Fig. S2A). We then purified short and long isoforms of AEBP2, along with the T1 and T2 mutants in complex with PRC2 (Fig. 2B). HMT assays showed that truncation of the N-terminal region of the long isoform alleviated its inhibitory effect on PRC2 activity, with both T1 and T2 mutants restoring H3K27 methylation to comparable levels (Fig. 2C). These results are consistent with previous findings implicating acidic motifs in mediating inhibition and further suggest that the broader N-terminal region contributes to this regulatory effect [25]. Together, these results demonstrate that the N-terminal region of the AEBP2 long isoform, including the DE-rich motifs, is required for its inhibitory effect on PRC2 activity.

### AEBP2 long isoform inhibits both EZH2 automethylation and H3K27 methylation through the DE-motif in its N-terminal

To test whether the DE-motifs affect PRC2–nucleosome interactions, we performed electrophoretic mobility shift assays using either mono-nucleosomes or di-nucleosomes as substrates. Both the long and short isoforms enhanced PRC2-nucleosome binding (Supplementary Fig. S2B and C), consistent with the previously reported role of the KR motif [7]. Complexes containing the short isoform exhibited slightly stronger nucleosome binding than those containing the long isoform, suggesting that the DE-rich region may modestly attenuate, but does not abolish, nucleosome association.

We then tested whether the N-terminal region of the AEBP2 long isoform may negatively regulate PRC2 catalytic activity via inhibition of EZH2 automethylation. To test this, we conducted methyltransferase assays measuring EZH2 automethylation, the major methylation at K514 [22–24]. While the short isoform stimulated EZH2 automethylation, the long isoform inhibited it in both nucleosome-free (Fig. 2D) and nucleosome-containing conditions (Supplementary Fig. S2D). Importantly, this inhibitory effect was lost upon truncation of the N-terminal region. Consistent with these findings, analysis of nuclear extracts from TKO cells rescued with the long isoform revealed reduced Ezh2-K514me3 levels compared to those expressing the short isoform (Fig. 2E), further supporting that the long isoform inhibits Ezh2 automethylation in a cellular context.

Together, these results indicate that the N-terminal region of the AEBP2 long isoform restrains PRC2 catalytic activity, through inhibition of EZH2 automethylation.

### Differential regulation of PRC2 recruitment and activity by AEBP2 isoforms

**Figure 3.**
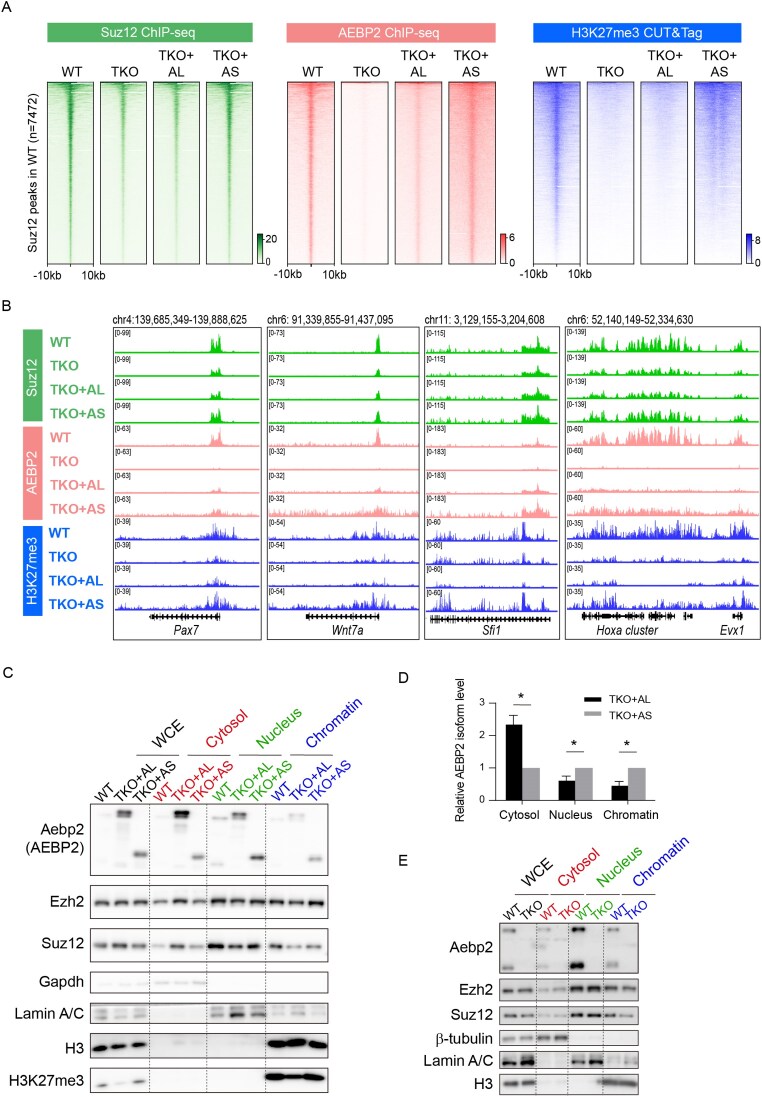
Isoform-specific modulation of PRC2 genomic recruitment and enzymatic activity by AEBP2. (**A**) Heatmaps showing spike-in normalized Suz12 ChIP-seq (left), AEBP2 ChIP-seq (middle), and H3K27me3 CUT&Tag (right) signals centered on WT Suz12-bound regions (*n* = 7472) in WT, TKO, and TKO mESCs rescued with either long (AL) or short (AS). (**B**) Genome browser views showing Suz12 ChIP-seq, AEBP2 ChIP-seq, and H3K27me3 CUT&Tag profiles at representative developmental loci (*Pax7, Wnt7a, Sfi1*, and *Hoxa* cluster genes). (**C**) Subcellular fractionation analysis showing the distribution of AEBP2 isoforms and PRC2 core components (Ezh2 and Suz12) across cytosolic, nuclear, and chromatin-bound fractions in WT, TKO + AL, and TKO + AS cells. Fractionation quality was assessed using Gapdh (cytosolic marker), Lamin A/C (nuclear marker), and histone H3 (chromatin marker). (**D**) Quantification of relative AEBP2 isoform levels across subcellular fractions is shown in (**C**). Data are presented as mean ± SD (*n* = 3). Statistical significance was determined using an unpaired two-tailed Student’s *t*-test; *P < .05. (**E**) Subcellular fractionation analysis of AEBP2 and PRC2 core components in WT and TKO cells across cytosolic, nuclear, and chromatin-bound fractions. Fractionation markers are shown as in panel (C), except that β-tubulin was used as the cytosolic marker.

We next examined how AEBP2 isoforms regulate PRC2 chromatin association and activity in a cellular context. Given that Mtf2 and Jarid2 are major mediators of PRC2 recruitment, and that the long isoform of AEBP2 exhibited a pronounced inhibitory effect under their depletion (Fig. 1C), we analyzed genome-wide localization of AEBP2 and Suz12 by ChIP-seq, together with H3K27me3 profiles by CUT&Tag in TKO mESCs lacking *Mtf2, Jarid2*, and *Aebp2*, rescued with either the long or short AEBP2 isoform (Fig. 3A and B).

Consistent with previous observations [27, 28], TKO cells exhibited reduced Suz12 binding and decreased H3K27me3 deposition at Polycomb-enriched (Suz12 peak centered) regions. Re-expression of the AEBP2 long isoform (TKO + AL) did not restore the PRC2 recruitment defect or H3K27me3 levels. In contrast, re-expression of the short isoform (TKO + AS) partially restored both Suz12 occupancy and H3K27me3 deposition. These findings indicate that the AEBP2 short isoform supports PRC2 chromatin association and activity, whereas the long isoform fails to restore PRC2 recruitment and H3K27me3 deposition in cells lacking Mtf2 and Jarid2.

This functional difference is accompanied by distinct subcellular localization patterns. The AEBP2 long isoform was predominantly enriched in the cytosolic fraction, and the short isoform was enriched in soluble nuclear and chromatin-bound fractions (Fig. 3C and D). Accordingly, PRC2 core components, including Ezh2 and Suz12, were enriched in the cytosolic fraction and reduced in the nucleus and chromatin-bound fraction in TKO and TKO + AL cells, whereas chromatin association was partially restored in TKO + AS cells (Fig. 3C and E).

Interestingly, at a subset of genomic regions, increased binding of the AEBP2 long isoform in TKO + AL cells was associated with reduced H3K27me3 despite little or no increase in Suz12 occupancy compared with TKO cells, as observed at the *Sfi1* and *Hoxa* cluster loci (Fig. 3B). These locus-specific patterns suggest that AEBP2-L is ineffective at restoring functional PRC2 activity on chromatin, consistent with our *in vitro* observations (Fig. 2).

Genome-wide bin-based analysis further confirmed these trends as well. The short isoform largely restored global H3K27me3 levels toward WT cells, whereas the AEBP2 long isoform failed to do so (Supplementary Fig. S3A–C). Signal intensities across these bins mirrored the patterns observed in heatmaps, with TKO + AL exhibiting the lowest H3K27me3 levels (Supplementary Fig. S3D). AEBP2 occupancy was also higher in TKO + AS than in TKO + AL (Supplementary Fig. S3E). SUZ12 binding was reduced in TKO cells relative to WT and was modestly increased by AEBP2-S rescue, whereas AEBP2-L did not restore SUZ12 occupancy to a similar extent (Supplementary Fig. S3F).

We further examined the relationship between AEBP2 gain and PRC2 activity at both the genome-wide level using 10-kb bins and at gene promoters defined as TSS ± 2 kb. Spearman correlation analysis using spike-in-normalized signals across all 10-kb bins revealed a clear positive association between AEBP2 gain and H3K27me3 increase in both WT and AEBP2-S rescue conditions (ρ ≈ 0.4), indicating coordinated PRC2 recruitment and catalytic activity. In contrast, this relationship was markedly weakened in the AEBP2-L condition (ρ ≈ −0.12), suggesting that AEBP2-L occupancy is uncoupled from H3K27me3 deposition (Supplementary Fig. S3G–I). Similar results were observed at promoter regions, where WT and AEBP2-S conditions showed positive correlations, whereas AEBP2-L showed only weak or no correlation (Supplementary Fig. S3J–L).

Together, these findings indicate that in the absence of canonical recruitment factors such as MTF2 and JARID2, the AEBP2 short isoform partially restores PRC2 chromatin recruitment and H3K27me3 deposition, whereas the long isoform fails to restore the PRC2 recruitment defect and is unable to support effective H3K27me3 deposition.

### The AEBP2 long isoform reduces H3K27me3 and derepresses genes

We next examined whether the reduction in H3K27me3 induced by the AEBP2 long isoform leads to transcriptional de-repression and changes in active chromatin marks. To this end, we performed RNA-seq together with CUT&Tag profiling of H3K4me3.

**Figure 4.**
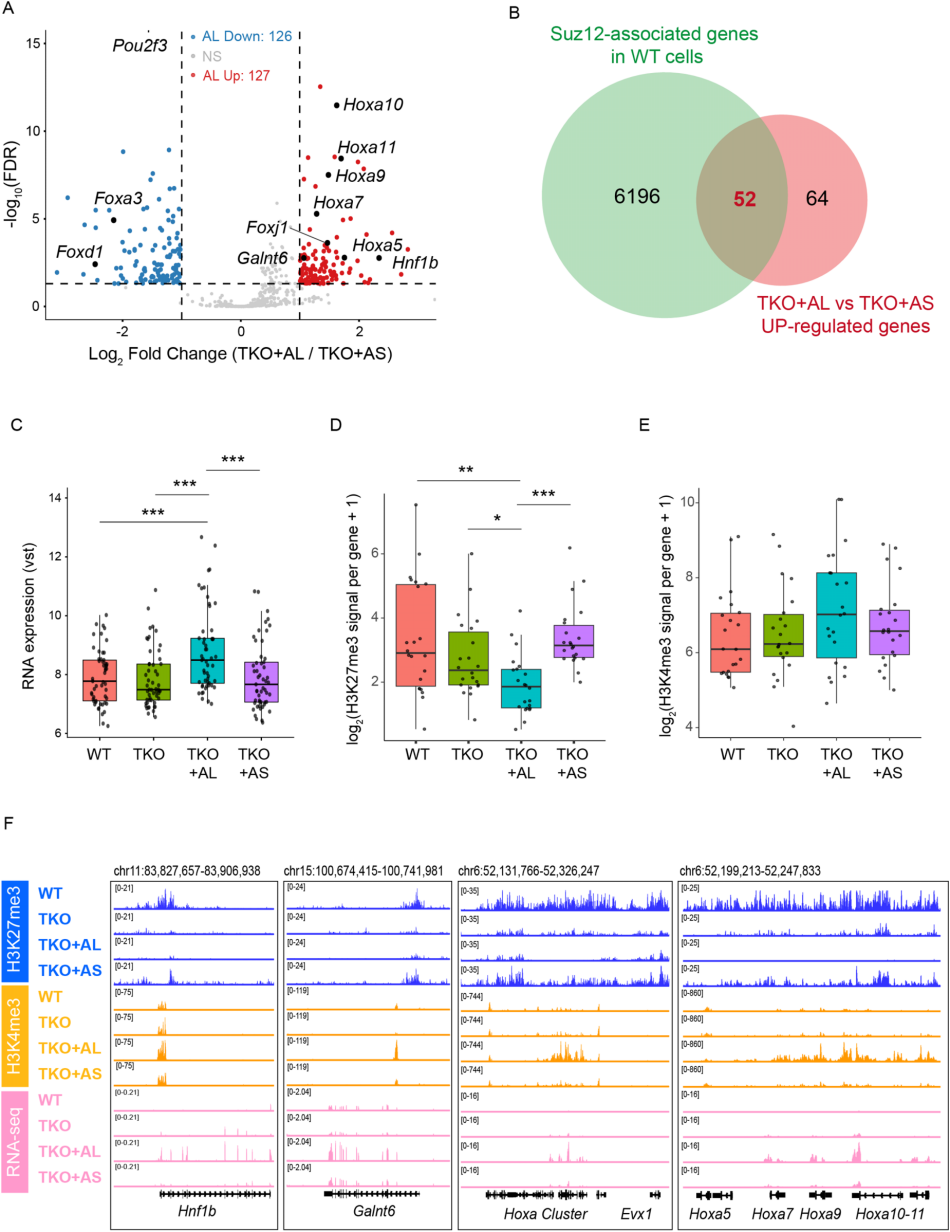
The AEBP2 long isoform reduces H3K27me3 and derepresses Polycomb target genes. (**A**) Volcano plot showing differential gene expression between TKO + AL and TKO + AS cells. Genes with an absolute log_2_-fold change >1 and FDR <0.05 are highlighted (red, upregulated in TKO + AL; blue, downregulated in TKO + AL). Selected representative developmental genes are labeled. (**B**) Venn diagram showing the overlap between genes upregulated in TKO + AL relative to TKO + AS and genes associated with WT Suz12 peaks. Suz12-associated genes are defined as those containing Suz12 peaks within ±2 kb of the TSS. A total of 52 overlapping genes were identified and used for subsequent analyses. Numbers indicate the size of each gene set and their overlap. (**C**) Box plot showing RNA expression levels (DESeq2-normalized vst counts) of 52 overlapping genes identified in panel (B). (**D**) Box plot showing H3K27me3 CUT&Tag signal (log_2_-transformed signal per gene + 1) among the 52 overlapping genes with detectable H3K27me3 signal, across the indicated conditions. (**E**) Box plot showing H3K4me3 CUT&Tag signal (log_2_-transformed signal per gene + 1) among the 52 overlapping genes with detectable H3K27me3 signal, across the indicated conditions. (**F**) Genome browser views showing H3K27me3, H3K4me3, and RNA-seq profiles across representative loci (*Hnf1b, Galnt6, Hoxa3*–*Hoxa11* cluster), with a detailed view of the *Hoxa5*–*Hoxa11* region, in WT, TKO, TKO + AL, and TKO + AS cells. Statistical significance in boxplots was determined using the Wilcoxon rank-sum test; *P < .05, **P < .01, ***P < .001.

Compared to TKO + AS cells, TKO + AL cells exhibited widespread transcriptional changes, including upregulation of developmental regulators such as the *Hoxa* cluster (Fig. 4A). Consistently, principal component analysis (PCA) of RNA-seq data showed that TKO + AL cells were the most transcriptionally distinct among the conditions (Supplementary Fig. S4A). In addition, a subset of genes, including *Foxa* family members and *Pou2f3*, was downregulated, reflecting secondary transcriptional effects (Fig. 4A). To identify putative direct PRC2 targets, we intersected genes upregulated in TKO+AL relative to TKO+AS with genes associated with WT Suz12 peaks, yielding a set of 52 candidate genes (Fig. 4B). Analysis of these genes revealed that TKO + AL cells exhibited the highest RNA expression levels, accompanied by reduced H3K27me3 and a modest increase in H3K4me3 compared to other conditions (Fig. 4C–E). Because this stringent overlap identified a relatively small set of candidate direct targets, we performed Gene Set Enrichment Analysis to assess PRC2-dependent transcriptional changes at a broader scale. Suz12 target genes identified in WT mESCs were used as the gene set for GSEA, and genes from the RNA-seq dataset were pre-ranked according to their log_2_-fold change between TKO + AL and TKO + AS cells. This analysis revealed that Suz12 target genes were significantly enriched toward the TKO+AL-upregulated end of the ranked gene list, with a leading-edge subset of 1719 genes (Supplementary Fig. S4B). We then examined RNA expression and H3K27me3 levels across expressed leading-edge genes after excluding genes with low expression. Consistent with the focused 52-gene analysis, TKO + AL cells showed the highest RNA expression and the lowest H3K27me3 levels across these leading-edge genes, supporting the conclusion that the AEBP2 long isoform promotes transcriptional derepression through reduced PRC2 catalytic activity (Supplementary Fig. S4C and D). Representative loci, including *Hnf1b, Galnt6*, and the *Hoxa* cluster, further illustrated this relationship, showing reduced H3K27me3 accompanied by increased H3K4me3 and transcriptional activation in TKO + AL cells (Fig. 4F).

Next, we investigated whether genes derepressed in TKO cells relative to WT due to reduced H3K27me3 can be repressed again by the stimulatory effect of the AEBP2 short isoform, given that TKO + AS cells largely restore H3K27me3 levels. To this end, we examined genes upregulated in TKO versus WT that overlap with WT SUZ12 peaks, identifying 162 genes (Supplementary Fig. S4E). Analysis of RNA expression revealed that although H3K27me3 levels were largely restored in TKO + AS cells, transcriptional repression of these genes was only partially rescued (Supplementary Fig. S4F and G). This partial rescue is also consistent with the PCA analysis, in which TKO + AS cells clustered more closely with TKO than with WT cells (Supplementary Fig. S4A). This incomplete rescue of gene repression may be explained by the absence of Mtf2 and Jarid2, and differences in the genomic distribution of restored H3K27me3 peaks, which in TKO + AS cells are more frequently located in intergenic and intronic regions rather than promoter or exon regions (Supplementary Fig. S4H).

Together, these results demonstrate that the AEBP2 long isoform promotes transcriptional derepression of PRC2 target genes, associated with reduced H3K27me3 and locus-specific increases in H3K4me3, thereby highlighting its inhibitory effect on PRC2 function in a cellular context.

### AEBP2 long isoform alone impairs early differentiation

**Figure 5.**
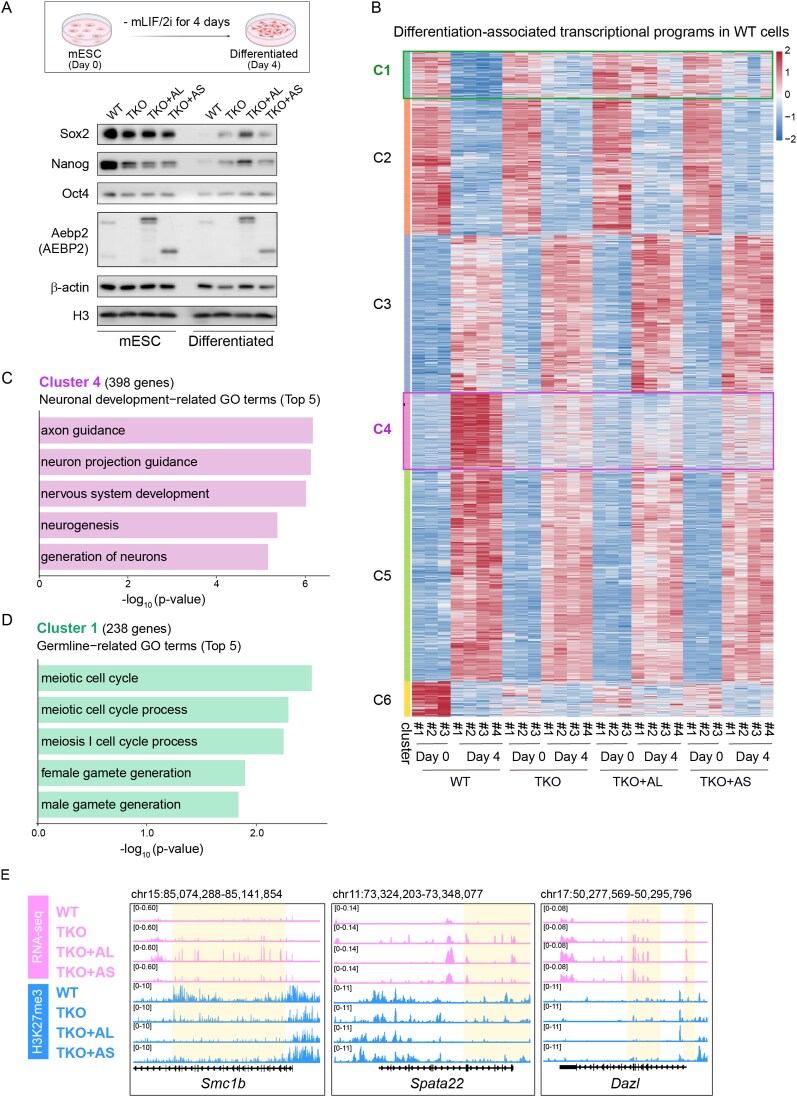
Lack of the AEBP2 short isoform results in impaired early differentiation-associated transcriptional programs. (**A**) Schematic representation of spontaneous differentiation of mESCs at day 0 and day 4 following removal of mLIF and 2i (top). Western blot analysis showing expression of pluripotency markers (Sox2, Nanog, and Oct4) and AEBP2 in WT, TKO + AL, and TKO + AS mESCs at day 0 and day 4 of spontaneous differentiation (bottom). β-actin and H3 serve as loading controls. Created in BioRender, Lee, C. (2026) https://BioRender.com/a0t5k2u. (**B**) K-means clustered heatmap of WT differentiation-associated transcriptional programs across all RNA-seq samples. WT differentiation signature genes were defined as genes differentially expressed between WT (day 4) and WT (day 0) using a threshold of FDR < 0.05 and |log_2_FC| > 2. Colored side bars indicate k-means clusters. “Cluster 1” and “Cluster 4” showed the most prominent transcriptional alterations in TKO + AL cells. (**C**) GO analysis of “Cluster 4” genes from panel (B) (*n* = 398), performed using DAVID GO Biological Process FAT (GO BP FAT). The top five neuronal development-related GO terms are shown. Bars indicate −log_10_ (*P*-value). (**D**) GO analysis of “Cluster 1” genes from panel (B) (*n* = 238), performed using DAVID GO BP FAT. The top five germline-related GO terms are shown. Bars indicate −log_10_ (*P*-value). (**E**) Genome browser views showing RNA-seq and H3K27me3 CUT&Tag signals at representative germline gene loci (*Smc1b, Spata22*, and *Dazl*).

To assess the impact of each AEBP2 isoform on early differentiation, we induced spontaneous differentiation of WT, TKO, TKO rescue cells with (AL or AS) by withdrawing LIF/2i and monitored the expression of pluripotency markers. Notably, TKO + AL cells maintained high levels of Sox2 and Nanog even after four days of differentiation (Fig. 5A and Supplementary Fig. S5A), indicating impaired early differentiation in these cells.

We next performed RNA-seq and H3K27me3 CUT&Tag in differentiated WT, TKO, TKO + AL, and TKO + AS cells to examine genome-wide transcriptional changes and chromatin states. The PCA analysis of all differentiated and undifferentiated cells showed that all samples shifted toward the differentiated state following LIF/2i withdrawal (Supplementary Fig. S5B).

To define the lineage characteristics of differentiation, we analyzed genes with decreased H3K27me3 and increased RNA expression (*n* = 218) in WT cells by comparing mESC and differentiated states following LIF/2i withdrawal (Supplementary Fig. S5C). GO analysis revealed enrichment for neuronal differentiation processes, indicating that WT cells preferentially adopt a neuronal lineage under these conditions (Supplementary Fig. S5D).

We then performed hierarchical clustering analysis of WT differentiation-associated transcriptional programs and identified gene clusters that were most strongly dysregulated in TKO + AL cells (Fig. 5B). Cluster 4, which was broadly reduced in TKO-derived differentiated cells compared with WT differentiated cells, was associated with neuronal development-related GO terms (Fig. 5C). This pattern suggests that the loss of major PRC2 accessory factors attenuates the normal differentiation-associated transcriptional program, with only a modest additional reduction in TKO + AL cells. In contrast, cluster 1 showed selective upregulation in TKO + AL cells, whereas it remained relatively repressed in WT, TKO, and TKO + AS cells. This cluster was enriched for germline-related GO terms (Fig. 5D), indicating that AEBP2-L permits aberrant activation of germline-associated transcriptional programs during differentiation. Consistent with this aberrant germline-associated transcriptional program, representative TKO + AL-upregulated germline genes, including *Smc1b, Spata22*, and *Dazl*, exhibited reduced H3K27me3 and increased RNA expression most prominently in TKO + AL cells (Fig. 5E). These transcriptional and chromatin changes were unlikely to be caused by higher AEBP2-L expression, as isoform-specific RNA quantification and immunoblotting showed that AEBP2-L and AEBP2-S were expressed at comparable levels (Supplementary Fig. S5E and Fig. 5A).

Together, these results demonstrate that the AEBP2 long isoform disrupts normal differentiation by altering PRC2-mediated repression, leading to aberrant activation of inappropriate lineage-associated gene programs while attenuating somatic lineage progression.

## Discussion

The activity of PRC2 is finely tuned by its accessory proteins, whose expression and function vary depending on developmental and cellular context. Most accessory proteins serve to stimulate PRC2 activity, including PHF1, MTF2, PHF19, JARID2, EPOP, and the short isoform of AEBP2 [2, 29, 30]. In contrast, the disease-relevant EZHIP (also known as CATACOMB) antagonizes PRC2 function in a manner similar to the H3K27M oncohistone [31–35].

In this study, we identified the long isoform of AEBP2 as a negative regulator of PRC2 activity, aligning with findings from a recently published study [25]. While the short isoform promotes PRC2 activity, the long isoform inhibits it through a unique DE-rich motif located at its N-terminus. Previous work from Mucha *et al.* demonstrated that the long isoform substantially inhibits PRC2 binding to DNA. However, this inhibitory effect is relatively modest in the nucleosome context shown from our study (Supplementary Fig. S3B and C) and a previous study, suggesting that the long isoform may preferentially inhibit PRC2 binding to DNA/chromatin in open chromatin regions rather than closed chromatin.

Importantly, extending beyond prior studies, we demonstrate that the long isoform, but not the short isoform, inhibits EZH2 automethylation, representing a key mechanistic insight. EZH2 automethylation at K510, K514, and K515 is known to regulate its catalytic activity by relieving autoinhibition and enabling H3K27 methylation [23, 24]. Our results show that the long isoform, via its DE-rich motif, suppresses EZH2 automethylation both *in vitro* and in cells (Fig. 2D and E, and Supplementary Fig. S2D), likely by interfering with access to the catalytic site. While the prior study [25] using histone H3 peptides reported no reduction in H3K27 methylation, these minimal substrates can readily access the catalytic pocket and therefore primarily reflect intrinsic enzymatic activity. In contrast, the native H3 tail engages multiple regions of PRC2, including the EZH2 CXC domain [36], and full catalytic activation requires automethylation-dependent displacement of the autoinhibitory loop from the catalytic site. Accordingly, our findings show that the long isoform inhibits EZH2 automethylation, thereby attenuating PRC2 activity either by directly limiting access to the catalytic site or indirectly by preventing automethylation and consequently reducing H3K27 methylation.

The GS-rich region located between the DE-motifs suggests that the N-terminal region of AEBP2 long isoform is highly flexible, which may explain why this segment was not resolved in prior structural studies. Such intrinsic flexibility could allow the N-terminus to sample multiple conformations and potentially interact with regions of EZH2 that include the automethylation loop [23, 24] and RNA-binding surfaces enriched in KR residues [37, 38], as well as with the KR-motif of AEBP2 itself [7], thereby contributing to reduced nucleosome or DNA binding, and negatively regulating the catalytic activity of PRC2. Consistent with this idea, the recent study showed that deletion or charge-reversal mutations within the N-terminal acidic region of AEBP2-L can relieve its inhibitory effect on PRC2 activity, supporting the functional importance of this region [23]. It will be important to determine whether the GS-rich region functions solely as a flexible linker or actively contributes to PRC2 inhibition, and whether the two DE-rich motifs have distinct or cooperative roles. Future mutational analyses, including deletion of the GS-rich region and mutants retaining DE1 while lacking DE2, together with structural approaches, will be required to define how individual elements of the N-terminal region of AEBP2-L cooperate to suppress EZH2 automethylation and PRC2 catalytic activity.

To dissect isoform-specific functions in cells, we employed *Mtf2*/*Jarid2*/*Aebp2*-TKO mESCs and reintroduced either the long or short AEBP2 isoform. This system provides a clearer functional readout than *Aebp2* single-knockout cells, where Mtf2 and Jarid2 can compensate for Aebp2 loss. In the TKO background, rescue with the long isoform results in reduced PRC2 activity and derepression of target genes, including *Hoxa* cluster genes. Notably, Hoxa9 is implicated in leukemogenesis [39], raising the possibility that differential expression of AEBP2 isoforms may have disease relevance. This warrants further investigation into isoform-specific expression patterns in leukemia.

All mESC experiments were conducted under 2i conditions, which are known to alter Polycomb organization and PRC2 distribution compared to serum culture. Although naïve conditions can influence chromatin binding and lineage repression [40], 2i remains a standard system for studying Polycomb function. Within this context, we consistently observe robust and reproducible effects of AEBP2 isoforms on PRC2 activity and chromatin association.

While the long isoform contains a conserved KR-motif that is shared with the short isoform and known to enhance nucleosome binding, it also harbors distinct DE-rich motifs that are essential for its inhibitory function. This functional duality of the AEBP2 long isoform, with KR-mediated chromatin tethering alongside DE-mediated catalytic inhibition, enables it to simultaneously recruit PRC2 to chromatin and restrain its catalytic activity. Such architecture likely evolved to provide tighter control over PRC2 activity, particularly in contexts where widespread H3K27 methylation must be restricted.

Consistent with this model, the expression of AEBP2 isoforms is dynamically regulated across developmental stages. The short isoform is predominantly expressed during early development, coinciding with high levels of PRC2 core components such as EZH2, EED, and SUZ12. During this stage, pluripotency must be preserved even as cells undergo rapid proliferation, and hence H3K27 methylation must be sustained following DNA replication. The abundant expression of the AEBP2 short isoform at this time is likely key in enhancing PRC2 activity to meet this biological requirement, highlighting a functional correlation between isoform expression and PRC2 catalytic activity. By contrast, terminally differentiated, non-dividing cells have much less requirement for PRC2 activity, congruent with the observed downregulation of PRC2 core components and parallel declines in accessory proteins expression [7, 41–43]. Notably, differentiated cells exhibit a marked loss of the AEBP2 short isoform, with sustained expression of the long isoform as the predominant variant in differentiated cells. This isoform switch, from an activator to an inhibitor of PRC2, reflects the reduced catalytic needs of the complex, suggesting that developmental stage-specific expression of AEBP2 isoforms provides a mechanism to dynamically modulate PRC2 activity according to cell state [8, 14].

H3K27me3 profiles in TKO cells rescued with AEBP2-S exhibited a broader, bimodal pattern compared with the sharp central enrichment observed in WT cells. Previous studies have suggested that PRC2 nucleation and spreading are mechanistically distinct processes that can be partially uncoupled [44]. In this context, the reduced central enrichment observed in TKO + AEBP2-S cells may reflect impaired PRC2 recruitment due to the absence of the major recruitment factors Mtf2 and Jarid2, whereas the broader flanking H3K27me3 signal may indicate relatively preserved spreading activity. Although the underlying mechanism remains unclear, these observations are consistent with the possibility that AEBP2-S contributes to the spreading phase of PRC2 activity, which may become more apparent when Mtf2- and Jarid2-mediated recruitment is compromised. More broadly, our findings are consistent with recent studies showing that regulatory domains within PRC2 subunits and other chromatin-modifying enzymes can influence recruitment, spreading, and catalytic regulation [45, 46]. In this context, AEBP2 isoforms may provide an additional layer of specificity by differentially modulating PRC2 chromatin binding, H3K27me3 spreading, and catalytic activity.

Specifically, this work reveals a finely balanced regulatory system through which differing isoforms encoded by the same gene can have antagonistic roles, exerting both activating and repressive effects on PRC2. This isoform-based regulation enables context-dependent tuning of PRC2 activity during development and differentiation, adding to the growing framework of alternative splicing and modular domain architecture as key strategies for achieving epigenetic precision.

## Supplementary Material

gkag694_Supplemental_File

## Data Availability

The sequencing data generated in this study have been deposited in the NCBI Gene Expression Omnibus under accession number GSE310209. Original gel images were uploaded to Zenodo: https://doi.org/10.5281/zenodo.20964805. This study did not generate original software or novel computational algorithms.
