# Supplementary material for: Opposing function of AEBP2 isoforms fine-tune PRC2 catalytic activity
Source: Nucleic Acids Res. 2026 Jul 9;54(13):gkag694. doi: 10.1093/nar/gkag694 (PMC13347268; doi:10.1093/nar/gkag694)
Supplement: gkag694_Supplemental_File [file gkag694_supplemental_file.pdf]

Supplementary Information

Supplementary Figure 1

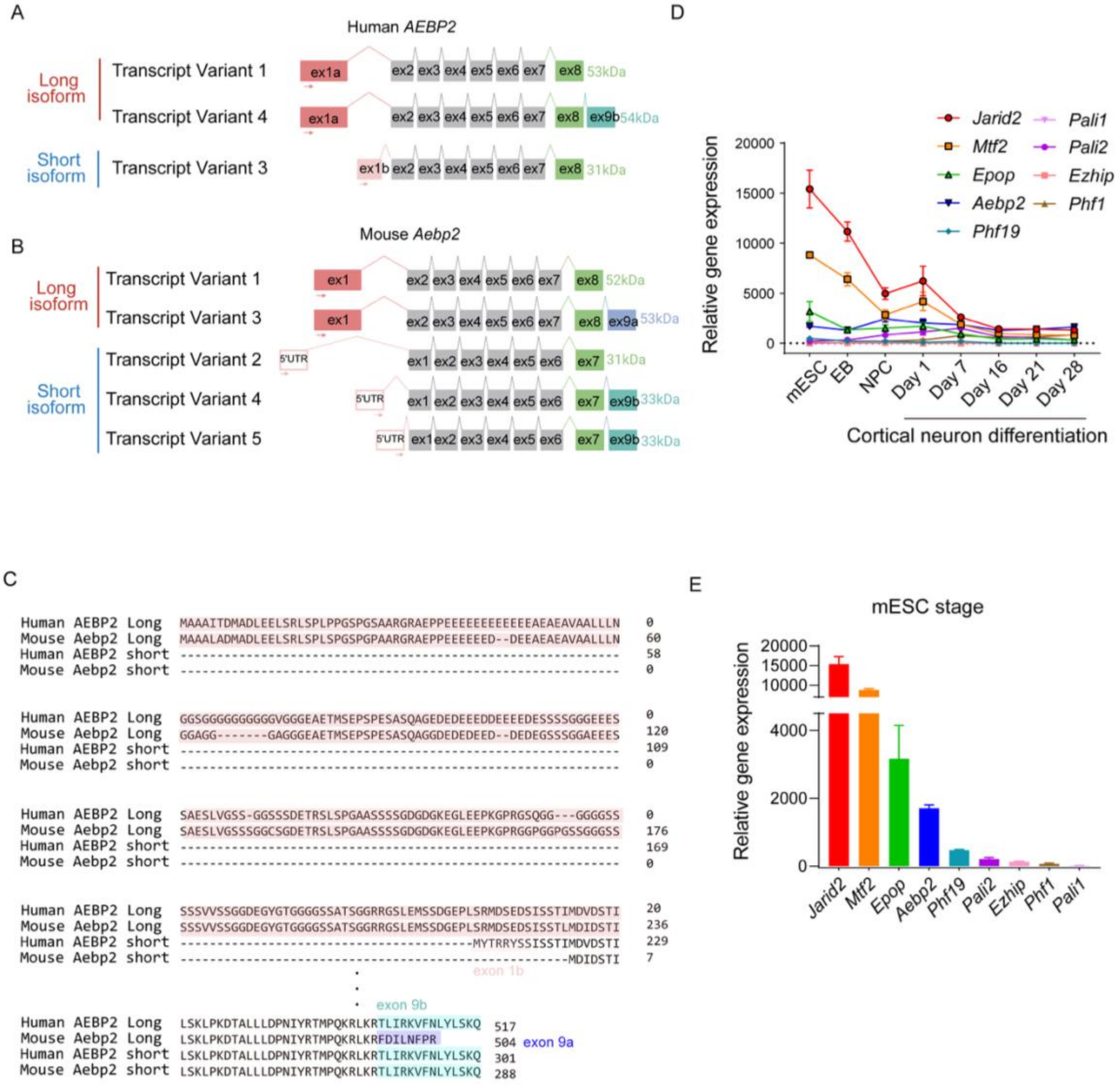

Supplementary Figure 1. Alternative transcription generates conserved AEBP2 isoforms with distinct expression relative to PRC2 accessory factors.

(A-B) Schematic diagrams of the human *AEBP2* (A) and mouse *Aebp2* (B) gene structures illustrating alternative transcription start sites that generate two distinct isoforms. Coding exons are shown as filled boxes, with isoform-specific exons highlighted to indicate isoform-specific regions. **(C)** Protein sequence alignment of the N-terminal and C-terminal regions of human AEBP2 and mouse Aebp2 isoforms. The long isoform possesses an extended N-terminal region encoded by exon 1a, which contains acidic, DE-rich motifs that are absent in the short isoform. Exons 9a and 9b are also shown, as they give rise to distinct transcript variants. **(D–E)** Gene expression dynamics of PRC2 accessory proteins during differentiation from mESCs to cortical glutamatergic neurons (D) and their expression levels at the mESC stage (E). Data was obtained from a previously published longitudinal RNA-seq dataset. Expression values represent RSEM-normalized counts, shown as mean ± s.d.

Supplementary Figure 2

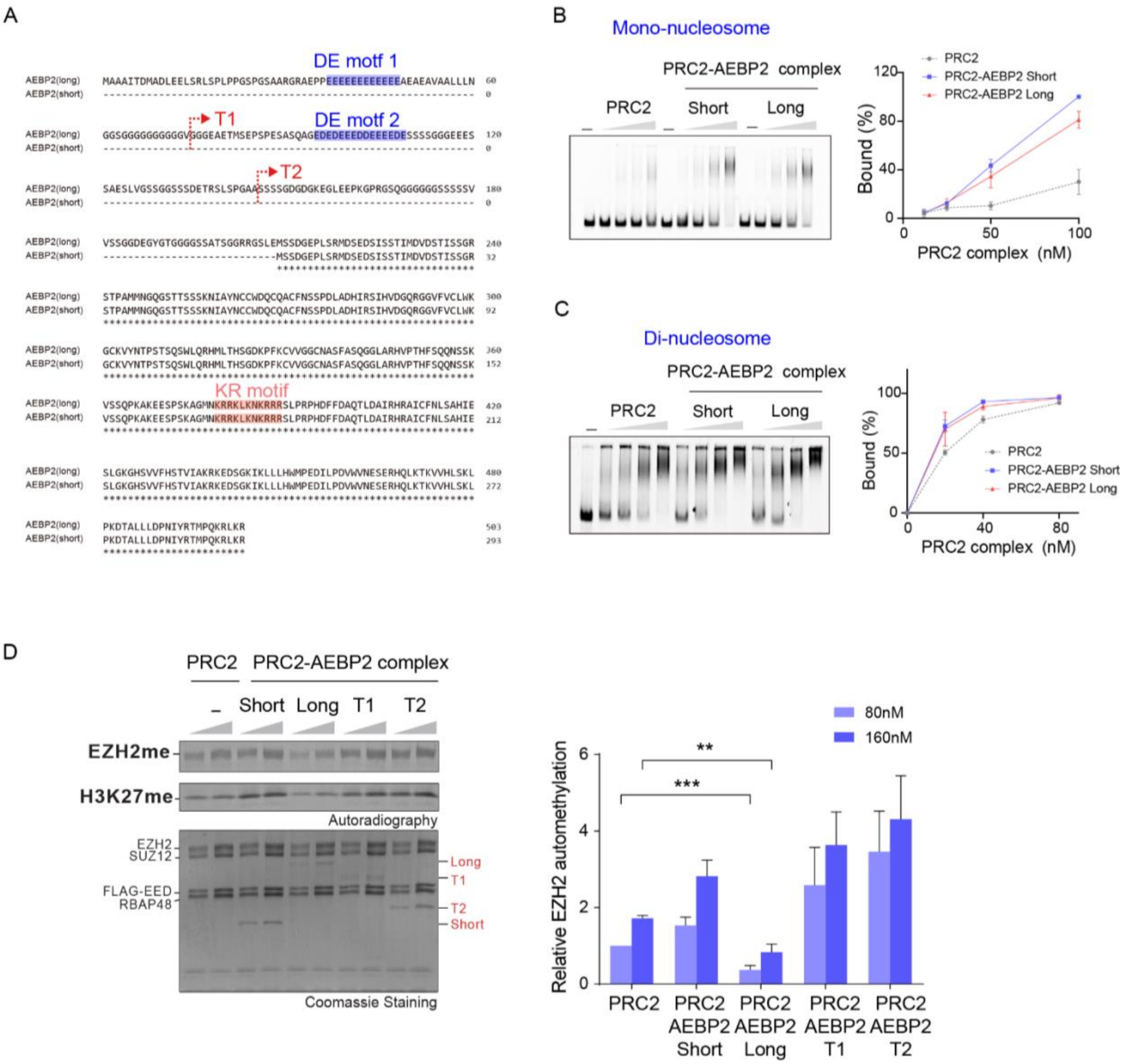

Supplementary Figure 2. The acidic DE-rich motifs in the N-terminal region of the AEBP2 long isoform inhibit PRC2 catalytic activity.

(A) Schematic representation of human AEBP2 long and short isoform protein sequences showing the two DE-rich motifs (blue) unique to the long isoform and the KR motif (red) conserved in both isoforms. Truncated mutants lacking either one (T1) or both (T2) DE-rich motifs were generated to assess the roles of these motifs in PRC2 regulation. **(B-C)** Electrophoretic mobility shift assays (EMSAs) using PRC2 complexes reconstituted with AEBP2 long or short variants, and either mono-nucleosomes (B) or di-nucleosomes (C) as substrates. Quantification of bound fractions (right) is shown across increasing PRC2 concentrations. Data are presented as mean ± s.d. (n = 3). **(D)** Histone methyltransferase assay containing PRC2 or PRC2-AEBP2 complexes (50, 100 nM) with ^{3}H-labeled S-adenosylmethionine (SAM) and di-nucleosomes (150 nM) (Top). Quantification of the relative amount of EZH2 automethylation after 60 min of incubation is shown (n = 3/data point) (Bottom). Statistical significance was determined using unpaired two-tailed Student's t-test; $^{**}p < 0.01$, $^{***}p < 0.001$.

Supplementary Figure 3

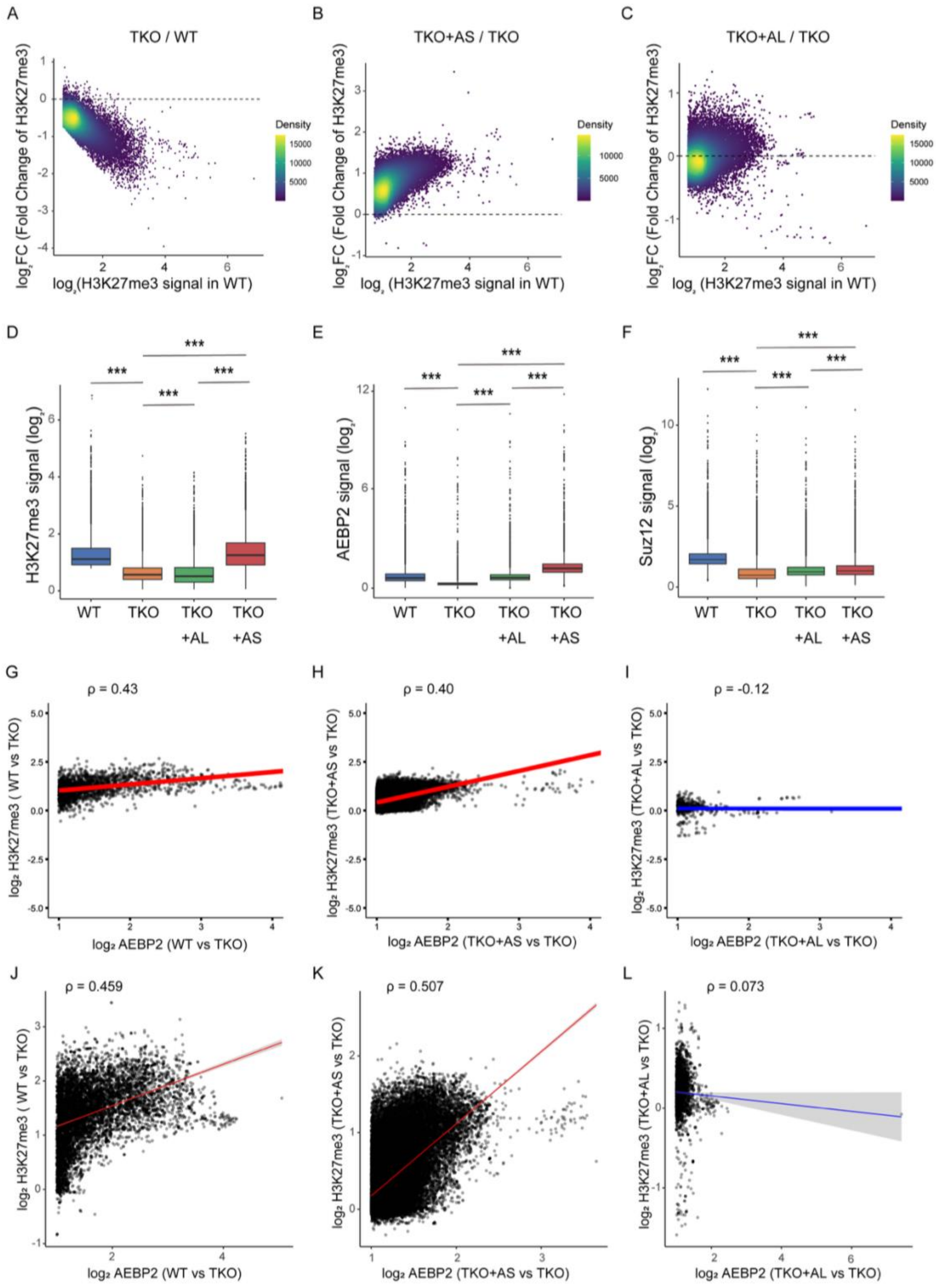

Supplementary Figure 3. Isoform-specific modulation of PRC2 genomic recruitment and enzymatic activity by AEBP2.

(A-C) Density scatter plots showing the relationship between H3K27me3 signal in WT cells (x-axis) and $\log_2$ Fold Change ($\log_2$FC) in the indicated conditions (y-axis) across 10-kb genomic bins. Comparisons include TKO versus WT (A), TKO+AS versus TKO (B), and TKO+AL versus TKO (C). Each point represents a 10-kb genomic bin within the upper 0.9 quantile of H3K27me3 signal in WT cells. **(D-F)** Box plots showing the distribution of (D) H3K27me3, (E) AEBP2, and (F) Suz12 signal intensities across the same 10-kb genomic bins. Signal values are shown as $\log_2$-transformed normalized counts. Statistical significance was assessed using the Wilcoxon rank-sum test (***$P < 0.001$). **(G-I)** Correlation between AEBP2 gain and H3K27me3 changes across 10-kb genomic bins. Each dot represents a genomic bin with increased AEBP2 signal relative to TKO, defined as $\log_2$ (AEBP2 condition/TKO + 1) > 1. The x-axis shows $\log_2$ AEBP2 enrichment relative to TKO, and the y-axis shows the corresponding $\log_2$ H3K27me3 change relative to TKO for WT (panel G), TKO+AS (panel H), and TKO+AL (panel I) conditions. Solid lines indicate linear regression fits. Spearman correlation coefficients (ρ) are shown for each comparison. **(J-L)** Correlation between AEBP2 gain and H3K27me3 changes at promoter regions (TSS ± 2 kb). Each dot represents a promoter bin with increased AEBP2 signal relative to TKO, defined as $\log_2$ (AEBP2 condition/TKO + 1) > 1. The x-axis shows $\log_2$ AEBP2 enrichment relative to TKO, and the y-axis shows the corresponding $\log_2$ H3K27me3 change relative to TKO for WT (panel J), TKO+AS (panel K), and TKO+AL (panel L) conditions. Solid lines indicate linear regression fits. Spearman correlation coefficients are shown for each comparison.

Supplementary Figure 4

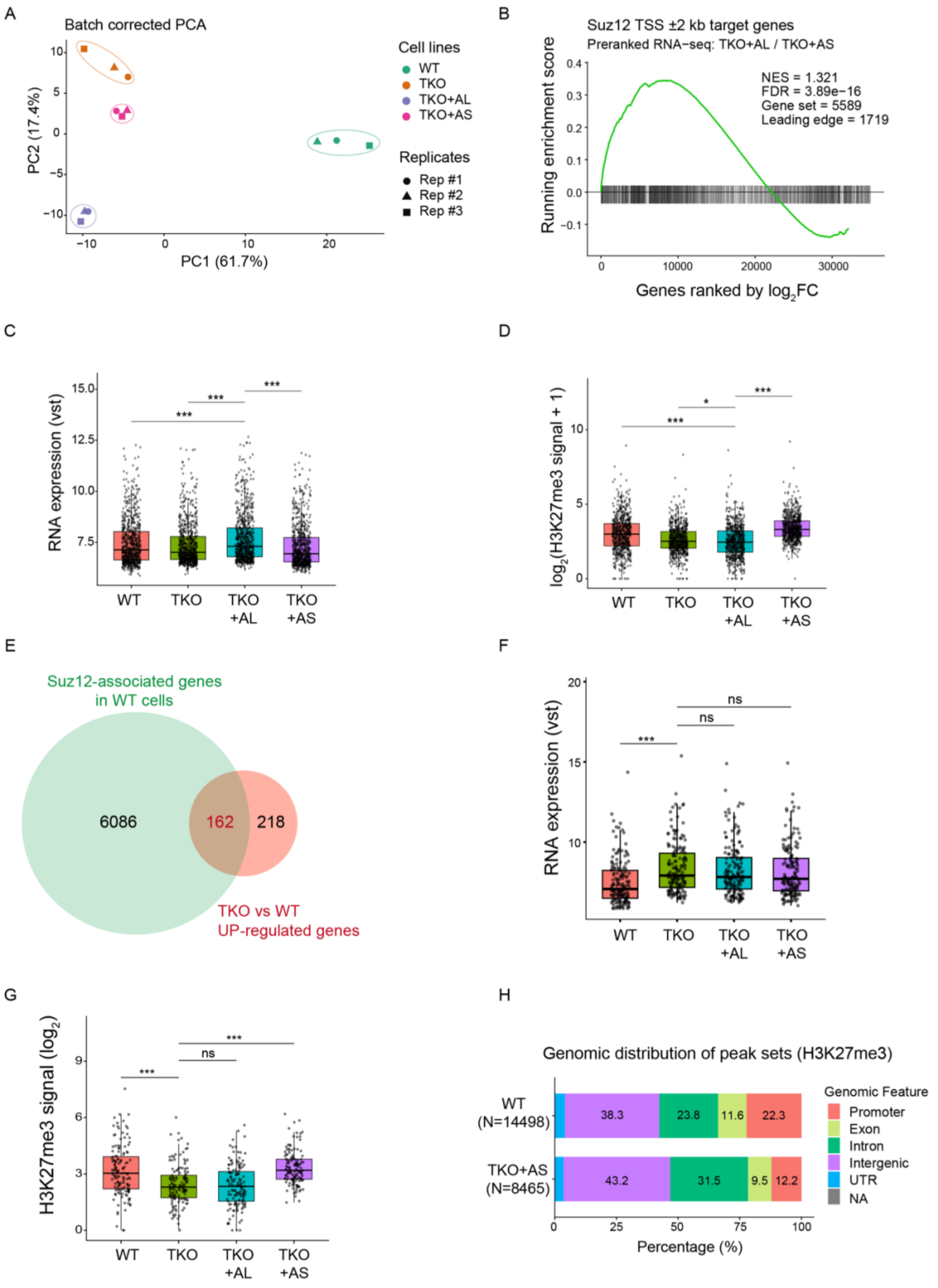

Supplementary Figure 4. AEBP2 short isoform largely restores PRC2 catalytic activity but only partially rescues gene repression.

(A) Batch-corrected PCA of RNA-seq profiles from WT, TKO, TKO+AL, and TKO+AS mESCs. Each point represents an individual biological replicate, with shapes indicating replicate number and colors indicating genotype. **(B)** Gene set enrichment analysis (GSEA) of Suz12 peak-associated genes in WT mESCs, defined as genes with Suz12 peaks within ±2 kb of the TSS, using RNA-seq genes pre-ranked by $\log_2$ fold-change between TKO+AL and TKO+AS. NES, FDR, gene set size, and leading-edge gene number are indicated. **(C)** RNA expression of Suz12 leading-edge genes from panel B after filtering for baseMean ≥ 10. Boxplots show variance-stabilized expression values averaged across replicates for each condition (n = 737 genes). Statistical comparisons are indicated above brackets. **(D)** H3K27me3 signal at expressed Suz12 leading-edge genes that overlap H3K27me3 peaks. Boxplots show $\log_2$ (H3K27me3 signal + 1) averaged across replicates for each condition (n = 676 genes). Statistical comparisons are indicated above brackets. **(E)** Venn diagram showing the overlap between genes upregulated in TKO versus WT and Suz12 target genes in WT mESCs, defined as genes with Suz12 peaks within ±2 kb of the TSS. Numbers indicate the size of each gene set and their overlap. **(F-G)** Box plot showing RNA expression levels (DESeq2-normalized vst counts) (F) and H3K27me3 CUT&Tag signal ($\log_2$-transformed signal per gene + 1) (G) for the overlapping gene set defined in panel E, across WT, TKO+AL, and TKO+AS conditions. **(H)** Stacked bar plot showing the genomic distribution of H3K27me3 peak sets in WT and TKO+AS cells across genomic features (promoter, exon, intron, intergenic, and UTR). Numbers indicate the percentage of peaks in each category. Statistical significance in boxplots was assessed using the Wilcoxon rank-sum test (**p < 0.01, ***p < 0.001).

Supplementary Figure 5

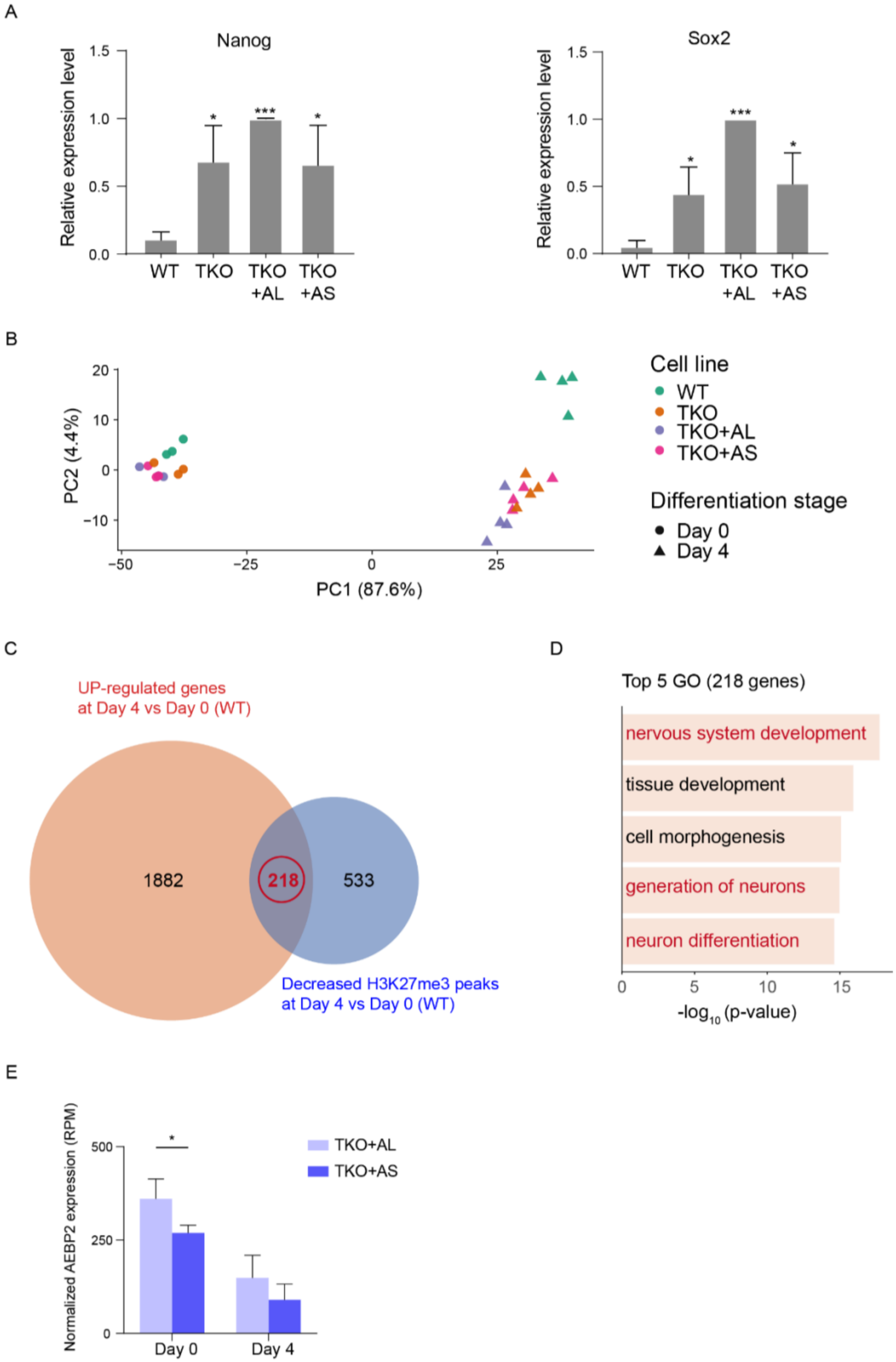

Supplementary Figure 5. Lack of the AEBP2 short isoform results in impaired early-stage differentiation.

(A) Quantification of Sox2 and Nanog protein levels from the Western blot shown in Figure 5A, normalized to β-actin. Data are presented as mean ± s.d. (n = 3). Statistical significance was determined using an unpaired two-tailed Student's *t*-test; *p < 0.05, ***p < 0.001. **(B)** PCA of RNA-seq samples from WT, TKO, TKO+AL, and TKO+AS cells at day 0 and day 4. Points represent biological replicates, colors indicate genotype, and shapes indicate differentiation state. **(C)** Venn diagram showing the overlap between genes with decreased H3K27me3 in WT Day 4 relative to WT Day 0 and genes with increased RNA expression. Upregulated genes were defined as $\log_2$FC > 2 with FDR < 0.05, whereas genes with reduced H3K27me3 were defined as $\log_2$FC < −1 with FDR < 0.1. **(D)** Gene Ontology (GO) biological process enrichment analysis of 218 overlapping genes between genes upregulated at Day 4 versus Day 0 and genes showing decreased H3K27me3 levels. Enrichment analysis was performed using the Database for Annotation, Visualization and Integrated Discovery (DAVID, v6.8; GOTERM_BP_FAT). The top five enriched GO terms are shown, ranked by $-\log_{10}$(p-value).

(E) Salmon-based isoform-level quantification of human AEBP2 rescue transcripts in the mouse RNA-seq samples. Human AEBP2 long isoform expression in TKO+AL and short isoform expression in TKO+AS were quantified using human AEBP2 reference sequences and normalized to RPM.
